# The Effect of Key Anthropometric and Biomechanics Variables Affecting the Lower Back Forces of Healthcare Workers

**DOI:** 10.3390/s23020658

**Published:** 2023-01-06

**Authors:** Xiaoxu Ji, Alexa Littman, Ranuki Onara Hettiarachchige, Davide Piovesan

**Affiliations:** Biomedical Engineering, Gannon University, Erie, PA 16541, USA

**Keywords:** healthcare, patient transfer, musculoskeletal disorder, spinal force, injury prevention

## Abstract

Wearable devices are becoming ubiquitous and can be used to better estimate postures and movements to reduce the risk of injuries. Thirty-three participants were recruited in this study to perform two daily repetitive patient transfer tasks while the full body movements were acquired using a set of magneto-inertial wearable devices. The use of wearable devices allowed for the estimation of the forces provoked on the lower back during the entire task performance. In postures where the forces exceeded the warning threshold found in the literature, healthcare workers were considered to have a greater risk of injury. Additionally, the maximum force exerted by each hand to avoid injury to the spinal column was also estimated. Knowing the key anthropometric variables associated with musculoskeletal disorders (MSDs) will enable engineers and researchers to design better assistive devices and injury prevention programs in diverse workplaces.

## 1. Introduction

As a result of the constant completion of strenuous tasks throughout the day, it is not uncommon for healthcare workers to experience musculoskeletal injuries. The most frequent of these injuries are in the upper and lower back, the neck, and the shoulders [1]. These injuries are a direct result of poor posture and repetitive tasks in awkward positions [1]. In 2018, a small survey of healthcare workers, including registered nurses and nursing assistants, reported that over 23,000 days were missed from work that year. Healthcare workers had the highest number of days away from work due to musculoskeletal disorders (MSDs) [2]. Although declining from 2019 to 2020, 2.7 million workplace injuries were still reported, equaling about 5.5 cases per 100 full-time equivalent workers [2]. These injuries can occur while accomplishing a variety of tasks; however, common strenuous tasks accomplished by healthcare workers include repositioning patients from various postures, such as transferring patients from lying in bed to sitting on a chair [3]. This repetitiveness of tasks, as well as working in awkward positions, results in overexertion and potential injuries [4,5].

Currently, approaches have been developed to prevent MSDs by intentionally making daily tasks for healthcare workers more ergonomically feasible. For example, the Occupational Safety and Health Administration (OSHA) is implementing hospital e-tool modules that address safe-patient handling. These modules review the proper way to handle patients, as well as ways to prevent MSDs while working and performing daily tasks [3]. Some assistive devices have also been designed to take the strain off the workers, such as mechanical wheelchairs as well as different lift assists [6,7,8,9,10]. According to the Centers for Disease Control (CDC), a three-tier intervention hierarchy is widely accepted for reducing, eliminating, or controlling workplace hazards, including ergonomic hazards [11]. However, to effectively determine the likelihood of MSDs in the task design phase, digital human modeling (DHM) technology has been widely used in several industries, including aviation, automotive, apparel design, military, energy, and industrial plants [12,13,14]. The forces exerted on the lower back of subjects could be adequately estimated by analyzing the adopted postures in task performance and the external forces applied by both hands [15].

While extremely useful, there are still many limitations in the use of DHM. For example, when predicting posture transitions by using the Task Simulation Builder (TSB) in JACK Siemens software (v9.0), significant differences were found between the estimated and actual human movement [16,17]. Such incongruencies directly resulted in a notable difference in forces exerted on the lumbar spine. Additionally, the time-consuming process of manually setting full body postures in DHM, as well as gathering the necessary information, communicating the details of the task to the engineers, and implementing the anthropometric information in the model, significantly constrains the development of dynamic simulations, as reported in [18].

This study presents healthcare workers’ lumbar spine force estimation using an integrated approach between DHM and a motion capture system. Our process prevents some of the limitations reported above. We used the Xsens advanced motion capture system to acquire human movements. The Xsens software (v2019) output can be interfaced with the JACK Siemens Software, allowing the DHM manikins to be synchronized in both software. The force output generated by the movements monitored using the Xsens was analyzed through JACK Siemens. This approach reduces the time of analysis and simulation. It allows for an accurate estimation of the forces exerted on the lower back of healthcare workers by replicating the intended movement within a dynamic simulation. This crucial process could effectively prevent workers from injury, as the size of the worker and the patient’s position may influence lumbar load [17]. Additionally, we estimated the maximum force exerted by each hand to avoid dangerous spinal compressive forces. Analyzing the correlation between anthropometric variables and the estimated spinal forces allows us to identify the key variables that could be conducive to MSDs. The proposed method can be used to design a work-related injury prevention program and optimize assistive devices in the workplace.

## 2. Materials and Methods

### 2.1. Participants and System Setup

The Xsens MVN Awinda system (Xsens 3D Motion Tracking Technology, Enschede, The Netherlands) was used to acquire full-body human movement. Thirty-three volunteers (16 females: average body height of 165.7 cm and body weight of 65.5 kg; 17 males: average body height of 180.6 cm and body weight of 83.4 kg) were involved in this study. Seventeen wearable inertial sensors were secured onto each participant according to the Xsens user manual. A customized digital human model was created in Xsens software (DHM_Xsens) using the anthropometric data of each participant and was used to record the actual human movement during task performance. This study has been approved by the university Institutional Review Board (GUIRB-2020-9-3567).

A unique feature of the JACK simulation tool (Siemens PLM software) is that it can seamlessly integrate with the Xsens MVN Analyze software. A second human model was created in JACK to represent each participant (DHM_JACK) to accomplish the integration. The skeletal roots of each DHM_Xsens were imported into JACK software to align with the corresponding skeletal segments of DHM_JACK by using its scaling feature. By doing so, the DHM_JACK would be driven by the DHM_Xsens to achieve an accurate representation of human movement.

### 2.2. Operational Tasks

Each participant was required to perform two daily repetitive patient transfer tasks. To consistency of task completion, participants had to follow the instruction provided in the UPMC Health System Nursing Assistant Orientation manual.

**Figure 1:**
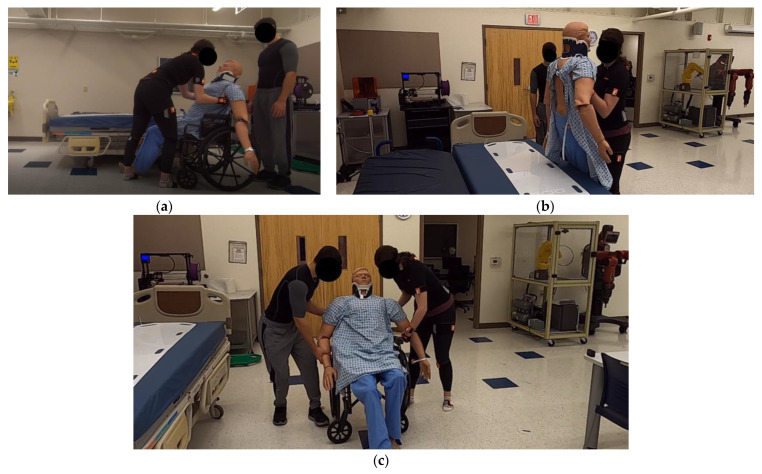
Task #1 transferring the patient mannequin from the wheelchair to the hospital bed. (**a**) Lifting the patient from the wheelchair. (**b**) Moving the patient over the hospital bed. Task #2 (**c**) Assisting the patient mannequin to stand from a seated position on the wheelchair.

Task #1: A 25 kg patient mannequin was seated in a wheelchair (Everest & Jennings Product, St. Louis, MO, USA). Each participant was required to transfer the mannequin from the wheelchair to the hospital bed (Hill-Rom Advanta, Hill-Rom Holdings, Inc., Chicago, IL, USA). This task involves lifting the patients and keeping them suspended above the wheelchair, then setting them down onto the bed, as shown in Figure 1. Each participant was required to perform the task four times at two different operational bed heights (higher height: 32.5 in; lower height: 25.5 in).

Task #2: Each participant was required to assist the patient’s mannequin to stand in the wheelchair. This task involves bending the participants’ trunks, placing their right hand under the patient’s arm, and straightening the participant’s trunk to lift the patient from the seated position in the wheelchair to the standing position in front of the wheelchair (Figure 1). Each participant was asked to perform the task four times to ensure the reliability of the results.

### 2.3. Data Analysis

#### 2.3.1. The Forces Exerted on the Lower Back

The compressive and Anterior/Posterior (A/P) shear force exerted on the 4th/5th lumbar spine (L4/L5) were evaluated using JACK ergonomics software by inputting the postures measured by the Xsens system together with the estimated magnitude and direction of force applied to each participant’s hand. The three specific poses that place healthcare workers at greater risk of getting MSDs were analyzed across the entire task. Such postures are assumed to provoke an excessive load on the participants’ lower backs.

**Figure 2:**
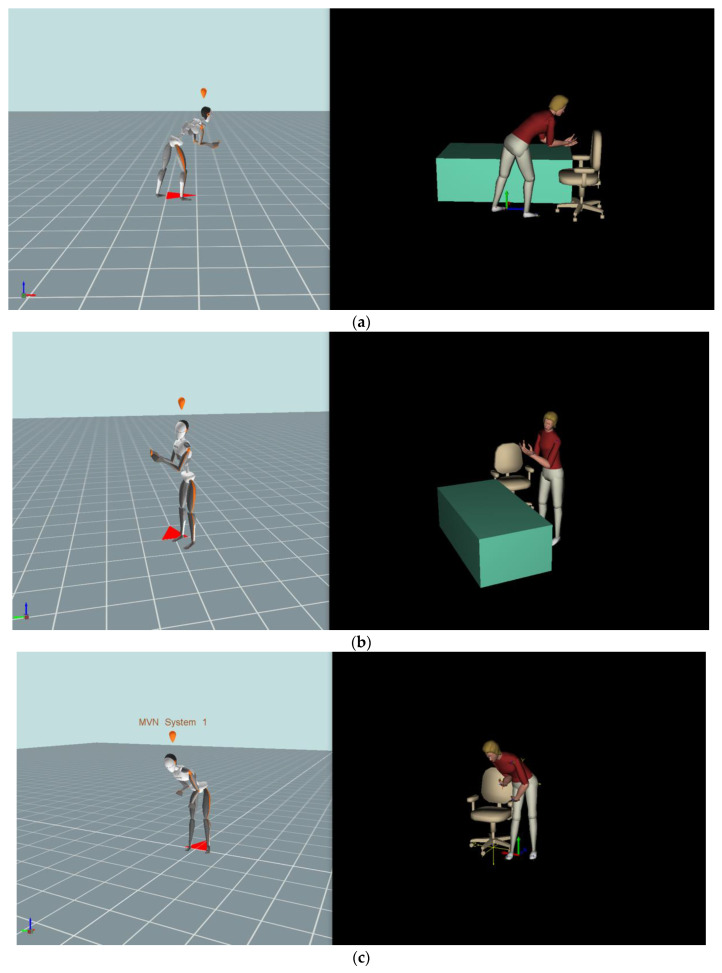
The three specific poses for the spinal forces analysis. The DHM_Xsens is on the left side to record the actual human movement. The DHM_JACK is on the right side, driven by the DHM_Xsens. (**a**) Pose #1. (**b**) Pose #2. (**c**) Pose #3.

Pose #1: In the task of transferring the patient from the wheelchair to the hospital bed, each participant needed to bend the knees and lean their upper body forward toward the patient, as shown in Figure 2a. Simultaneously, their arms were extended, and the hands were placed under the armpits of the patient to achieve the lifting effort. The peak spinal load was detected as the patient’s mannequin was suspended above the wheelchair. The force distributed to each hand of the participant was approximately 125 N (25 kg × 9.8/h), where h = 2 hands. The direction of the force applied by each hand was vertically upward. Although the support from the patient’s feet could alleviate the loads exerted by both hands, the estimated 125 N could still be used as input to reveal the participants’ adopted postures, which may significantly influence the spinal forces exerted by the lower back.

Pose #2: In the task of transferring the patient from the wheelchair to the bed, the participants began to move the patient over the hospital bed. Each participant stood close to the patient’s mannequin, flexed their elbows, and placed their hands under the patient’s arms to support them, as shown in Figure 2b. The force distributed to each hand was approximately 125 N, and the direction of the applied force was vertical and upward as well. The larger spinal load was estimated as the mannequin touched the edge of the hospital bed, inducing the participants’ trunk and hip flexion. Similarly, we could still use 125 N as a reference to reaffirm the correlation between the anthropometric variables and the spinal load as described above.

Pose #3: A two-person lift task to help the patient stand from a seated position in the wheelchair. This pose involves the participants bending their trunks to gain a stable base when lifting the patient. Each participant (on the patient’s left side) and the research assistant (on the right) exerted a lifting force by placing their dominant right hand under the patient’s arm, as shown in Figure 2c. Considering that each participant’s left hand slightly supported the mannequin’s forearm, the applied force on the left hand is assumed to be negligible. Hence, the force distributed to the right hand of each participant is assumed to be 125 N, and its direction is vertically upward.

#### 2.3.2. The Maximum Estimated Hand Force

The National Institute for Occupational Safety and Health (NIOSH) has determined that the safety threshold limit on the lower back compressive load is 3400 N [19]. The force on the lower back depends on the applied hand force and the posture adopted by the workers. To avoid healthcare workers’ injuries because of their daily repetitive tasks, it is essential to understand the maximum estimated hand force at the aforementioned three specific postures. Given the versatility of our dynamic simulations, the applied hand force was adjusted until the spinal compressive load reached 3400 N. These maximum hand forces will greatly help healthcare workers understand the safety limits of hand loads as they exert a lifting effort.

### 2.4. Statistical Analysis

A *t*-test was performed for each task to compare spinal forces and joint angles across different operational levels and genders. The statistically significant level was set at 0.05. To identify the individual factors influencing the spinal load, we calculated the correlation coefficient between body height, body weight, and anatomical joint angles with respect to the spinal load.

## 3. Results

### 3.1. Force Analysis

#### 3.1.1. Pose #1 in Task #1: Lift the Patient Mannequin from the Wheelchair

**Figure 3:**
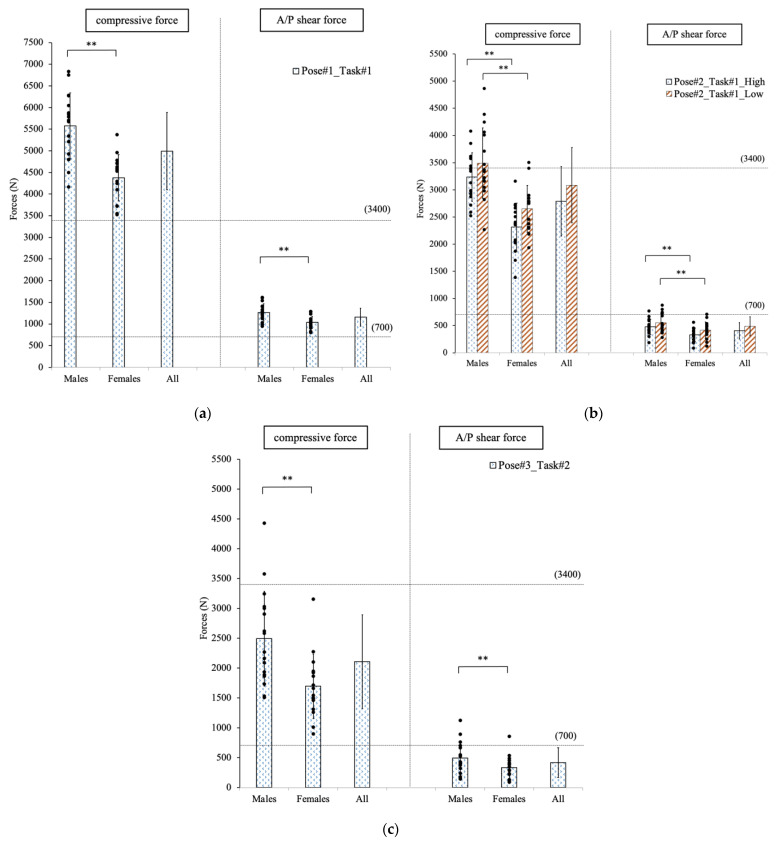
The forces exposed on the lower back of participants were analyzed at three specific poses. (**a**) Pose #1 in Task #1. (**b**) Pose #2 in Task #1. (**c**) Pose #3 in Task #2. ** indicated a significant difference between genders.

Figure 3a shows that the L4/L5 spinal forces were significantly different (*p* < 0.001) between the females and males as they lifted the patient mannequin from the wheelchair by flexing the trunk and simultaneously extending their arms, as shown in Figure 3a. The compressive lower backload of females and males were 4374.6 N and 5576.7 N, while the A/P shear forces were 1037.5 N and 1266.9 N, respectively. The spinal load on all participants was larger than the safety recommendation of 3400 N for the compressive force [19] and 700 N for the A/P Shear force [20].

To avoid the risk of injury on the lower back (3400 N safety threshold), females should not exceed a maximum force of 85.8 N at each hand, which was twice the force that males should exert not to injure themselves (42.7 N). There was a significant difference (*p* < 0.001) between genders, which is predictable given the larger body weight and moment arm males need to support.

#### 3.1.2. Pose #2 in Task #1: Transfer the Patient Mannequin to the Hospital Bed

As the participants transferred the mannequin from the wheelchair to the edge of the hospital bed (Figure 3b), the spinal forces were not significantly different (*p* > 0.05) at two operational heights. However, the spinal load on males and females was significantly different. At the higher operational height, the average spinal compressive force for males was 3235.8 N compared to 2312.7 N for females (*p* < 0.001). At the lower operational height, the forces were 3490.8 N for males and 2649.2 N and females, respectively (*p* < 0.001). For the A/P shear force, the spinal loads on the males and females were 476.1 N and 330.4 N at the higher height (*p* = 0.004) and 555.6 N and 417.8 N at the lower height (*p* = 0.02).

For the analysis of estimated hand force, there was no significant difference (*p* = 0.07) between two different operational heights for all participants. In addition, at this specific pose, there was no significant difference between genders at the higher operational height. However, the difference was significant at the lower operational height with a *p*-value of 0.0002 (males: 126.1 N; females: 173.1 N).

#### 3.1.3. Pose #3 in Task #2: Lift the Patient Mannequin from Sitting to Standing from a Wheelchair

When comparing genders, the spinal loads were significantly different, as shown in Figure 3c. For the compressive force, the *p*-value was 0.002 (males: 2491.9 N; females: 1694.5 N), and for the A/P shear force, the *p*-value was 0.04 (males: 485.2 N; females: 333.4 N).

Males need to exert a maximum force of 260.1 N on each hand to reach the recommended 3400 N safety threshold limit, whereas females need to exert 397.8 N with each hand. The difference was statistically significant (*p* = 0.0014).

### 3.2. Joint Angle Analysis

The two joint centers (Root and Spine#1) in JACK DHM are defined below:

- The root is the center of two greater trochanters.
- Spine #1 is the center of two posterior superior iliac spines (PSIS), which is at the waist level of spine L5.

Accordingly, the trunk flexion-extension movement is defined as the angular displacement in the sagittal plane between two vectors (1. From Acromion to Spine #1; 2. From Spine #1 to Root). As well as the hip flexion-extension movement is defined as the angular displacement between another two vectors (1. From Spine #1 to Root; 2. From Root to Knee).

Assuming that the 0° of trunk and hips are at a neutral posture, the positive and negative values for the trunk and hip angular displacements indicate the movement of flexion and extension, individually.

#### 3.2.1. Trunk Angles

**Figure 4:**
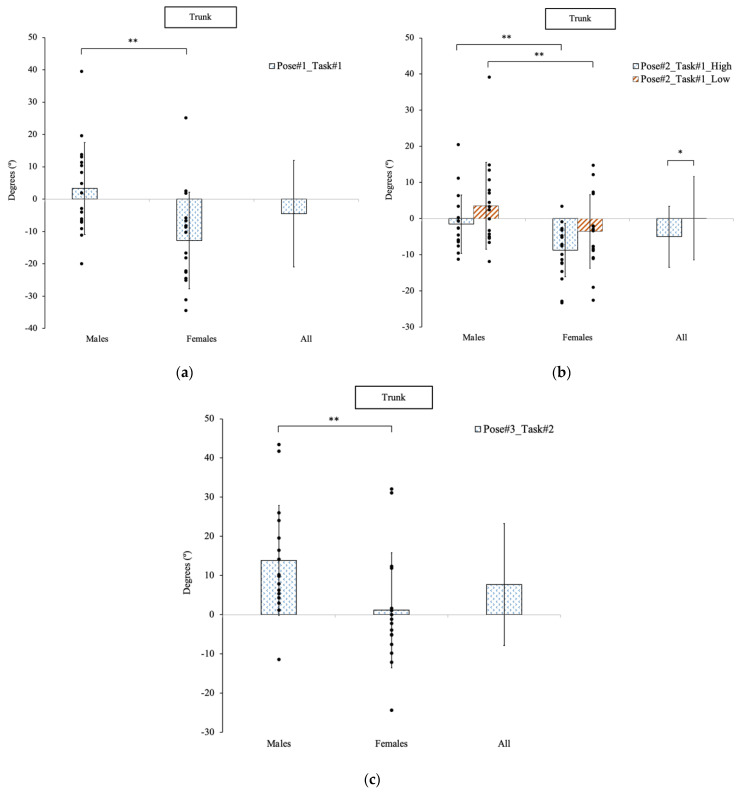
The trunk movement of participants to complete two patient transfer tasks at three specific poses. (**a**) Pose #1 in Task #1. (**b**) Pose #2 in Task #1. (**c**) Pose #3 in Task #2. * indicated a significant difference between the two heights. ** indicated a significant difference between genders.

The trunk angular displacement was significantly different between males and females at all three poses. The trunk angular displacement to lift the patient mannequin from the wheelchair at Pose #1 in Task #1 (Figure 4a) was 3.3° (flexion) for the males, while the females executed a −12.8° (extension) with the *p*-value of 0.003. At Pose #2 in Task #1 (Figure 4b), the males executed a −1.5° (extension), and the females assumed a −8.7° (extension) trunk angular displacement to transfer the patient mannequin to the hospital bed at the higher operational height (*p* = 0.012), and 3.5° (flexion) and −3.5° (extension) for the males and females at the lower operational height (*p* = 0.04). At Pose #3 (Figure 4c) in Task #2, the males averaged a 13.9° (flexion) and the females a 1.2° (flexion) trunk angular displacement to lift the patient mannequin from sitting in the wheelchair to standing with a *p*-value of 0.02.

Additionally, at Pose #2 in Task #1, the trunk angular displacement was significantly different (*p* = 0.04) between the two different operational heights for all participants. The total average trunk displacement was −5.0° (extension) at the higher height and 0.1° (extension) at the lower height.

#### 3.2.2. Hip Angles

**Figure 5:**
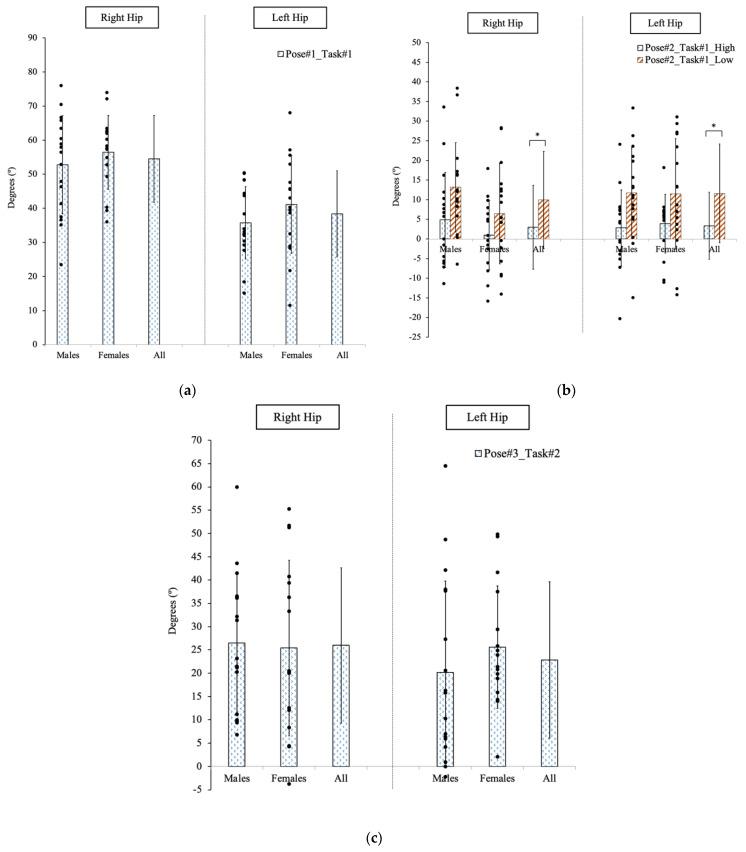
The hip movement of participants to complete two patient transfer tasks at three specific poses. (**a**) Pose #1 in Task #1. (**b**) Pose #2 in Task #1. (**c**) Pose #3 in Task #2. * indicated a significant difference between the two heights.

For the comparison of hip angular displacement, as shown in Figure 5, the only significant difference existed at Pose #2 in Task #1 between the two different operational heights when participants transferred the patient mannequin to the hospital bed. The *p*-values were 0.02 and 0.003 for the right hip and left hip, respectively. However, this difference did not exist in the comparison between genders (*p* > 0.05).

**Figure 6:**
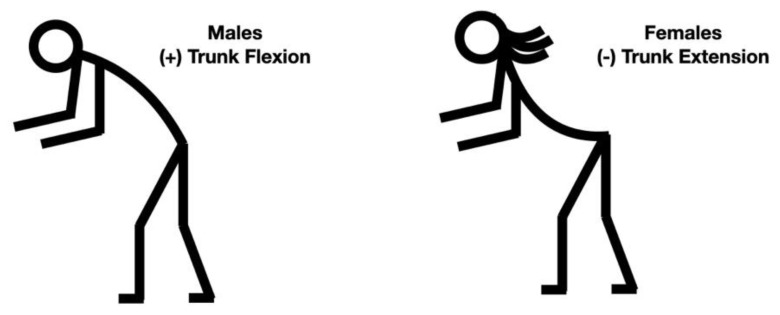
The preferred poses that males and females adopted during task performance. The positive trunk angles indicate flexion, and the negative values indicate flexion.

The difference in trunk movement between males and females is shown in Figure 6 when they adopted poses to lift a patient. The males preferred to flex their trunks, but the females preferred to extend their trunks during two patient transfer tasks.

### 3.3. Correlation Analysis

The anthropometric variable “body weight” was highly correlated to the compressive lower back load with r = 0.67 at Pose #1 in Task #1, r = 0.61 at Pose #2 in Task #1 at the higher operational height, r = 0.65 at Pose #2 in Task #1 at the lower height. A moderate correlation r = 0.53 was found at Pose #3 in Task #2. Furthermore, there was a high correlation between the body weight and A/P shear force with r = 0.70 at Pose #1 in Task #1, and a moderate correlation at other poses with the r-values of 0.45, 0.52, and 0.46, respectively.

The body height had a high correlation to the compressive and A/P shear forces at Pose #1 in Task #1 with the r = 0.86 and r = 0.85, respectively. At other poses, the correlations were high for the compressive force and moderated for the A/P shear force. At Pose #2, Task #1 in the higher height, r = 0.60 and 0.35 for the compressive force and A/P shear force, while at the lower height, r = 0.70 and r = 0.51. At Pose #3 in Task #2, we found r = 0.68 between body weight and compressive load and r = 0.55 between body weight and A/P shear load.

The trunk angular displacement was correlated to the compressive and A/P shear forces with the r values of 0.55 and 0.46 at Pose #1 in Task #1, as well as 0.73 and 0.64 at Pose #3 in Task #2. However, at Pose #2 in Task #1, the trunk movement was only correlated to the compressive force with r = 0.47 at the higher operational height.

The hip angular displacement had a moderate correlation with the A/P shear force at Pose #2 in Task #1 with r values of 0.52 (right hip) and 0.51 (left hip) at the higher height. However, at the lower height, the hip movement was not only highly correlated to the A/P shear force (right hip: 0.80; left hip: 0.73) but it was also correlated to the compressive force (right hip: 0.63; left hip: 0.43). Moreover, the right hip angular displacement was moderately correlated to the compressive and A/P shear forces with r = 0.51 and r = 0.64 at Pose #3 in Task #2.

A summary of the correlation coefficients between body height, body weight, and anatomical joint angles with respect to both compressive and A/P loads are listed in Table 1.

## 4. Discussion

We successfully evaluated the lower back load in participants performing patient transfer tasks common in healthcare. The examined tasks included transferring a patient from a wheelchair to a hospital bed and assisting the patient in standing from a wheelchair. According to OSHA guidelines [19], we estimated the maximum load exerted by each participant’s hand to avoid the risk of injuring their lower back. We highlighted the impact of key anthropometric and biomechanics variables on lower back forces at specific postures, which varies according to participants’ size and gender.

The proposed statistical analysis highlighted that the trunk and hip angular displacements led to spinal load magnitude changes given the hand’s exerted load. For example, as the participants increased their trunk and hip flexion, the forces imposed on the lower back were significantly increased. The hip and torso flexion increases the moment arm of the external load with respect to the lower back. Consequently, extensor moments in the erector spinae and lower back muscles are also increased [16,21]. The Lifting and Material Handling (UNC) resource [22] can provide proper lifting techniques to avoid MSDs or back injuries from daily repetitive patient transfer tasks. Suggestions include keeping the back as straight as possible and bending the knees to use the lower body strength to lift patients/objects. A straighter back allows for the core muscles to be engaged, increasing the stiffness of the lower back. Taller healthcare workers are at the highest risk during patient transfer activities because of their considerable trunk flexion, resulting in a sizeable compressive force on their lower back. Hence, adjusting the operational height according to the caregiver’s body height is essential to reduce the injury risk effectively.

For Pose #2 in Task #1, when the participants transferred the patient mannequin from the wheelchair to the hospital bed, the average trunk flexion was increased by 5°, and the hip flexion was increased by 8° as the operational height was changed from 32.5 into 25.5 in. The relatively small change in trunk flexion compared to hip flexion was due to the posture adopted by the participants. A lower operational height forced the participant to hold the patient mannequin closer to their upper body while performing the task, which constrained their trunk movement. Hence, at the lower operational height, hip flexion was the primary factor having a high correlation with the spinal load. However, at Pose #3 in Task #2, only the right hip angle was highly correlated with the forces at the lower back. Indeed, all participants stood on the left side of the patient mannequin to complete the sit-to-stand task from the wheelchair. Given that the participants slightly leaned toward their right side while simultaneously extending their left hip as they exerted a lifting effort, no significant difference in the left hip movement was observed.

Body weight is another key factor that also led to a significant difference in spinal load between genders. Notice that the lower-back load of males was greater than the females at all three specific postures. This result confirms the conclusion in [19], indicating that as the body weight supported by the lower trunk increases, the spinal load increases consequentially.

We estimated the maximum hand force the participant could exert at all three specific poses, which induced a 3400 N compressive force at the lumbar spine. This load places healthcare workers at a greater risk of getting MSDs. When participants started to transfer the patient mannequin from the wheelchair to the hospital bed (Pose #1 in Task #1), the estimated maximum exerted force was 85.8 N per hand for females and 42.7 N per hand for males. For the posture assumed, a 17.2 kg load will expose the females to injury risk, and a mere 8.5 kg load will expose the males. Although the muscle strength between genders is different [23], the males prefer to have larger trunk flexion than females in a lifting task, which leads to a significantly larger risk of injury for males caregiver.

Similarly, at Pose #2 in Task #1, when the participants transferred the patient mannequin to the hospital bed, nearly all males were exposed to a spinal compressive force higher than 3400 N with a 25 kg lifting load. Females would be exposed to the risk at an estimated load of 36 kg (180 N per hand). At Pose #3 in Task #2 when the participants assisted the patient mannequin from a seated position to a standing position, although the spinal forces were less than 3400 N for both males and females, the lifting load was only 25 kg. Yet, as the load increases to 52 kg, the males would be exposed to a lower back risk. Females would be exposed to injury if the load was 80 kg. Hence, distributing the load using an assisting device, such as those described in [8], would be ideal. Furthermore, trunk flexion should be reduced by getting closer to the patients and by bending the knees to transfer/lift the patients using full-body strength. Such a strategy should always be encouraged to avoid injury from daily repetitive tasks.

## 5. Conclusions

Posture control is essential for healthcare workers to avoid the risk of injury. The spinal loads of the thirty-three participants were successfully estimated during two daily repetitive patient transfer tasks. The hand force and a set of key anthropometric variables, including body height, weight, trunk movement, and hip movement, can determine the magnitude of the lower back load. The use of wearable sensors measuring the motions of humans in conjunction with DHM Technology to model the force at at-risk joints accurately can potentially bring benefits to healthcare, sport, and well-being.

In this study, we analyzed the task of moving a patient mannequin from a wheelchair to a bed. Considering that a small difference in the postures adopted by subjects will lead to a significant difference in loads exposed on the lower back, more common daily patient transfer tasks should be included. For example, it is necessary to analyze the task of moving the patient mannequin from a bed to a wheelchair and some tasks involving boosting the patient up in bed. It will benefit healthcare workers to fully understand how to avoid injuries during each of the tasks via extremely controlling the lifting load and the adopted postures.

## Figures and Tables

**Table 1:** The correlation coefficients for both compressive and A/P loads. Those values that are larger than 0.50 are highlighted in bold. “Comp” represents compressive force. “A/P” represents A/P shear force.

Variable vs. Force		Body Height	Body Weight	Hip Angles	Trunk Angles
Task1 pose1	Comp	**0.86**	**0.67**	0.16(R)/0.12(L)	**0.55**
	A/P	**0.85**	**0.70**	0.32(R)/0.10(L)	0.46
Task 1 pose 2 level high	Comp	**0.60**	**0.61**	0.36(R)/0.24(L)	0.47
	A/P	0.35	0.45	**0.52**(R)/**0.51**(L)	0.20
Task 1 pose 2 level low	Comp	**0.70**	**0.65**	**0.63**(R)/0.43(L)	0.10
	A/P	**0.51**	**0.52**	**0.80**(R)/**0.73**(L)	0.20
Task 2 Pose 3	Comp	**0.68**	**0.53**	**0.51**(R)/0.08(L)	**0.73**
	A/P	**0.55**	0.46	**0.64**(R)/0.34(L)	**0.64**
